# Moderate hypercapnia may not contribute to postoperative delirium in patients undergoing bronchoscopic intervention

**DOI:** 10.1097/MD.0000000000015906

**Published:** 2019-05-31

**Authors:** Qinghao Cheng, Lei Li, Mingyuan Yang, Lei Sun, Renjiao Li, Rui Huang, Jun Ma

**Affiliations:** a Department of Anesthesiology, Emergency General Hospital; b Department of Obstetrics and Gynecology, Emergency General Hospital; c Center for Anesthesiology, Beijing Anzhen Hospital, Capital Medical University, Beijing, China.

**Keywords:** bronchoscope intervention, hypercapnia, postoperative delirium

## Abstract

This study aimed to investigate the risk factors and whether acute hypercapnia contributes to postoperative delirium (POD) during bronchoscopic intervention under general anesthesia or deep sedation.

A prospective study was conducted with 119 consecutive patients who had undergone bronchoscopic intervention between February 2016 and December 2016 at the Emergency General Hospital.

Twenty-eight patients (23.8%) were diagnosed with POD. The patients were divided into 2 groups: the POD (n = 28) and the control group (n = 91). The mean age of the POD group was higher than that of the control group (*P* < .01). All the blood gas values, PaCO_2_ (*P* < .01), PaO_2_ (*P* < .01), and PH (*P* < .01), were significantly different. Multivariate analyses revealed that age (*P* < .01), operation duration (*P* = .01), and PO_2_ (*P* = .01) were independent predictive factors of POD, while hypercapnia (*P* = .54) was established as not being a predictive factor of POD.

Age, operation duration, and PO_2_ were determined as independent predictive factors of POD, whereas moderate hypercapnia is not likely to contribute to POD in patients undergoing bronchoscopic intervention. Clinical Trial Registration Identifier: ChiCTR-POC-15007483.

## 1 Introduction

Postoperative delirium (POD) has impacted 20% to 30% of patients who have undergone surgery,^[1,2]^ in addition to being associated with increased additional hospital care, institutional discharge, and re-hospitalization within 30 days of discharge.^[3]^ Based on past research, the preoperative risk factors of POD were preoperative cognitive impairment, heart failure, and age.^[4]^ Additionally, operational factors including hypercapnia and intraoperative hypotension are consequential to the development of POD.^[5]^

Bronchoscopic intervention has emerged as an alternative treatment that can reopen the airway and remove intraluminar malignancy, which would in return substantially improve patients’ quality of life.^[6,7]^ Bronchoscopic intervention that includes stenting, cryocanalization, electrocautery, microdebrider, and endobronchial ultrasound,^[8,9]^ has been typically carried out under general anesthesia or deep sedation. Common pathways have emerged as an intractable issue for both anesthesiologists and endoscopists.^[10]^ Airway stenosis due to bronchoscopy, and hypoventilation due to analgesic agents, can give rise to hypoxemia and hypercapnia during a bronchoscopic intervention.

The extensive use of permissive hypercapnia had been practiced for safeguarding the lungs in patients with chronic obstructive pulmonary disease (COPD), particularly in those receiving mechanical ventilation in the intensive care unit. As revealed by an animal study, moderate hypercapnia was noted to have a neuroprotective effect due to increase in cerebral perfusion and cerebral metabolic rate of oxygen (CMRO_2_) in rats.^[11]^ In clinical treatments, acute therapeutic hypercapnia during bronchoscopic intervention and thoracic surgery have been implemented with no serious consequences, in addition to enhancing the patients’ safety.^[12,13]^ However, a review has shown an association between hypercapnia and POD, and its impact has been quite unclear.^[5]^

In this research, we have performed an evaluation of the risk factors of POD and determined if acute hypercapnia contributes to its development during bronchoscopic intervention performed under general anesthesia or deep sedation.

## 2 Materials and methods

### 2.1 Study design and patient objectives

A total of 119 patients who were undergoing bronchoscopic interventions from February 2016 to December 2016 were recruited for this study. The research was approved by the ethics committee of the Emergency General Hospital in Beijing, China (No. K14–27). All the patients or their closest relatives signed an informed consent form before the initiation of the study protocol.

All bronchoscopic interventions were carried out by experienced endoscopists using electric flexible (Pentax, Japan) or rigid bronchoscopes. The inclusion criteria were

(i) age, ≤70 years;
(ii) duration of operation, between 30 and 120 minutes;
(iii) airway obstruction, <90%; and
(iv) patient literacy.

The exclusion criteria were

(i) history of cerebrovascular disease (e.g., stroke and heart surgery);
(ii) drug and alcohol usage (>1 drink/day);
(iii) illiteracy (incapable of participating in the assessment); and
(iv) diagnosed with hypoxia or hypercapnia prior to intervention.

### 2.2 Anesthetic settings and maintenance

The patients underwent an electrocardiogram (ECG), pulse oximetry, and bispectral index (BIS) after entering the operating room. Percutaneous application of CO_2_ and analyses of the arterial blood gases (ABG) were carried out during the procedure. The choice of anesthesia was dependent on the complexity of the surgery.

Remifentanil and propofol were used for deep sedation. Remifentanil (1 μg/kg) was intermittently injected and continuously infused (0.10–0.15 μg/kg·min) in accordance to the patient's vital signs during the procedure. Propofol (1 mg/kg) was administered 2 minutes after injecting remifentanil, followed by continuous infusion (30–50 μg/kg·min). The Observer's Assessment of Ramsay Sedation Scale (RSS) was maintained between 3 and 4.^[14]^ During the procedure, the patients retained spontaneous breathing, and lidocaine was administered with the spray-as-you-go technique.

General anesthesia was induced using fast-recovery drugs, namely, propofol, remifentanil, and rocuronium. Intravenous infusions of propofol and remifentanil were performed to maintain a deep level of anesthesia. Additionally, mechanical ventilation was performed using jet ventilation (Jiangxi Teli Medical Instruments, China). The respiratory rate was between 16 and 30 bpm, and the driving pressure was 3 kg/cm^2^ during the course of the intervention.^[13]^

When the SpO_2_ values were <90% or PaCO_2_ values were >100 mmHg, the endoscopists would stop the procedure and perform manual ventilation. When the PaCO_2_ value lowered to <80 mmHg, the procedure was resumed.

### 2.3 Assessment

POD was assessed in all the patients in the morning, from day 1 to 3 of the surgery, based on the criteria according to the D*iagnostic and* S*tatistical* M*anual of* M*ental* D*isorders* IV (DSM-IV), followed by evaluation of the scores. However, if the patient exhibited abnormal behavior, mood, consciousness, cognition, or sleep, the DSM assessment was carried out immediately, as specified in the DSM-IV criteria.^[15]^ Delirium was assessed by a mental health professional. The DSM-IV assessment took approximately 30 minutes when administered in a quiet environment with only the subject and investigator present.

### 2.4 Statistical analysis

Data analysis was performed with the SPSS software version 23.0 (SPSS Inc., Chicago, IL). The data were presented as mean ± standard deviation. The differences of the measurable data between the 2 groups were compared using a *t* test. Additionally, Chi-square test was used for countable data between the 2 groups. Furthermore, impact factors and POD were analyzed by logistic regression.

## 3 Results

### 3.1 Characteristics of patients in both groups

Among the 119 patients who had undergone bronchoscopic intervention, 23.5% (n = 28) were diagnosed with POD after a 3-day period. Significant differences in sex ratio, education, New York Heart Association (NYHA) classification, coronary heart disease, diabetes mellitus, hypertension, type of anesthesia, or pathology between the 2 groups were not observed (Table 1). The mean age of the POD group was higher than that of the control group (*P* < .01) (Table 1).

**Table 1:**
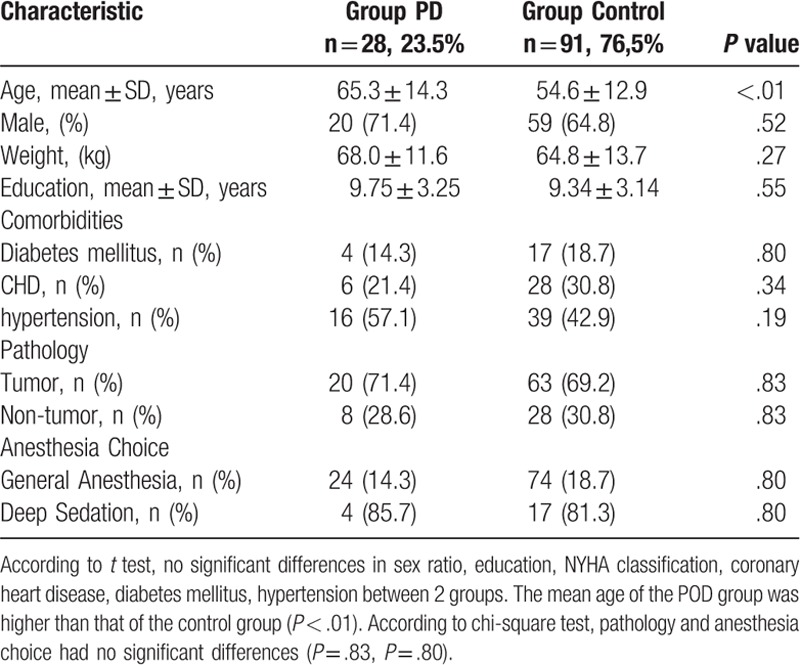
Comparison of preoperative patient characteristics.

All the blood gas values, procedure, and recovery time have been presented in Table 2. Significant differences in PaCO_2_ (*P* < .01), PaO_2_ (*P* < .01), and PH (*P* < .01) between both the groups were observed. However, significant differences in the potassium, glucose, and lactic acid contents of blood were not observed. Recovery time was faster in the control group than in the POD group (*P* = .01). The duration of the procedure was longer in the POD group, when compared with the control group (*P* < .01).

**Table 2:**
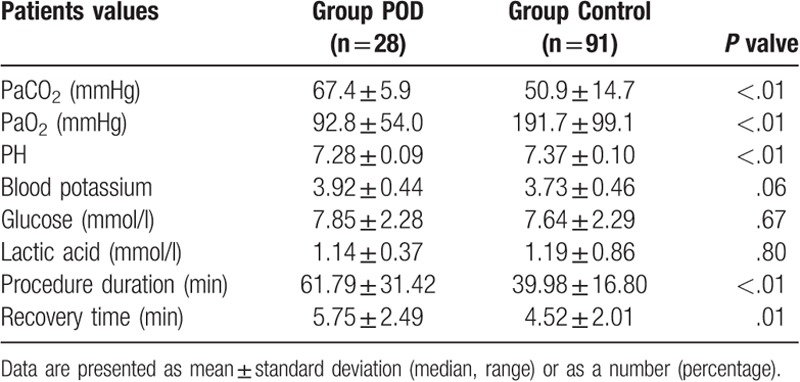
Blood gas values and procedure values.

### 3.2 Predictive factors for complications

For the identification of the predictive factors of POD (patient feathers, blood gas values, procedure, and recovery time), multivariable regression analysis was carried out, and age (*P* < .01), duration of operation (*P* = .01), and PO_2_ (*P* = .04) were determined as the independent predictive factors of POD. The PO_2_ was noted to have negative correlation with POD, whereas hypercapnia (*P* = .54) was established as not being a predictive factor of POD (Table 3).

**Table 3:**
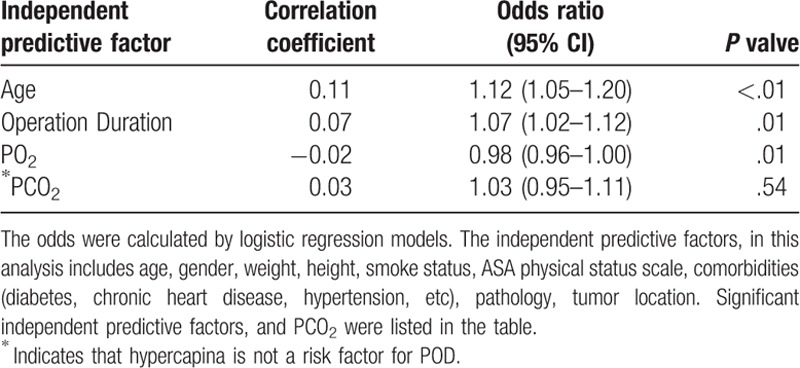
Independent predictive factors for postoperative delirium.

## 4 Discussion

POD is considered a key issue that accounts for a substantial part of the economic burden in perioperative settings, the incidence of which is dependent on the age of the patient and the type of surgery.^[16,17]^ The data from our study indicated that the short-term incidence rate of POD in patients undergoing bronchoscopic intervention was 23.5% in the first week. According to our research, education, chronic disease status, type of anesthesia, and type of surgery had no significance on the differences between the two groups, in contrast with the reports from other reviews.^[5,16,17]^ This is possibly due to the use of the same anesthetics in both the groups (propofol and remifentanil), and because long-acting narcotic drugs were not used.

In this research, the blood gas values revealed that the recovery time of the POD group was greater than that of the control group. The PaCO_2_ in the POD group (67.4 ± 5.9 mmHg) were higher than that of the control group (50.9 ± 14.7 mmHg), and the PaO_2_ in the POD group (92.8 ± 54.0 mmHg) was lower than that of the control group (191.7 ± 99.1 mmHg). The deficient blood values suggested that the POD group had hypoventilation due to the highly complex multiple bronchoscopic interventions as well as airway stenosis. In comparison with the control group, the POD group showed higher PaCO_2_ values and lower PaO_2_ values in the *t* test, implying poor characters and hypoventilation in the POD group.

Multivariable regression analysis highlighted that age (*P* < .01), operation duration (*P* = .01), and PO_2_ (*P* = .01) were independent predictive factors of POD during bronchoscopic intervention. However, hypercapnia (*P* = .54) was determined as not a predictive factor of POD. Past researches have also revealed that mild hypercapnia did not delay anesthesia recovery and the patients did not exhibit any neurologic deficits. Additionally, a study had demonstrated the neuroprotective effect of mild hypercapnia in rat models.^[11]^ However, a previous study had shown hypercapnia as an independent factor of POD; although the main reason for hypercapnia was not provided.^[5]^

Hypercapnia is regarded as having both favorable–unfavorable effects; mild hypercapnia is likely to progressively increase cerebral blood flow (CBF) and CMRO_2_, but (PaCO_2_: >100 mmHg) is likely to cause brain injury by the aggravation of brain edema, and thus worsening the brain's condition. In particular, severe hypercapnia with hypoxia lowered CBF, in addition to causing CMRO_2_ formalism attenuates neurovascular coupling.^[18–20]^ In the current research, when PaCO_2_ was >100 mmHg or SpO_2_ was <90%, the intervention was discontinued and mechanical ventilation was performed to improve the patient's condition. Acute and transient hypercapnia <100 mmHg was not a POD risk factor, whereas arterial oxygen desaturation was prevented in the patients receiving bronchoscopic intervention.

This research had a number of limitations. Firstly, this is a prospective study; moreover, the CO_2_ levels were obtained from an ABG analysis. Blood specimens were obtained using a transcutaneous monitoring system and surgical condition. Secondly, the levels of PaCO_2_ and PaO_2_ were limited in this study. Therefore, we were not capable of determining the values of severe hypercapnia with hypoxia. Thirdly, our conclusion was obtained from the patients undergoing bronchoscopic interventions. Thus, this conclusion is not recommended for other surgeries.

In conclusion, mild and moderate hypercapnia may not contribute to POD in patients undergoing bronchoscopic interventions. Finally, as mentioned previously, conclusive cognitive effects of permissive hypercapnia in clinical situations require further research.

## Author contributions

**Conceptualization:** Lei Sun, Rui Huang.

**Data curation:** Qinghao Cheng, Renjiao Li.

**Formal analysis:** Renjiao Li, Jun Ma.

**Funding acquisition:** Jun Ma.

**Investigation:** Qinghao Cheng, Lei Sun, Renjiao Li.

**Methodology:** Lei Li, Mingyuan Yang, Rui Huang, Jun Ma.

**Project administration:** Lei Li, Mingyuan Yang, Lei Sun, Jun Ma.

**Resources:** Mingyuan Yang, Jun Ma.

**Software:** Rui Huang.

**Supervision:** Lei Sun, Jun Ma.

**Validation:** Renjiao Li.

**Writing – original draft:** Qinghao Cheng, Rui Huang.

**Writing – review & editing:** Mingyuan Yang.
